# Optimal use of radiotherapy in the definitive treatment of non-bulky IB–IIA cervical cancer: A population-based long-term survival analysis

**DOI:** 10.1371/journal.pone.0253649

**Published:** 2021-06-24

**Authors:** Yu Jin Lim, Han Na Lee

**Affiliations:** 1 Department of Radiation Oncology, Kyung Hee University Medical Center, Kyung Hee University College of Medicine, Seoul, South Korea; 2 Department of Radiology, Kyung Hee University Hospital at Gangdong, Kyung Hee University College of Medicine, Seoul, South Korea; Women’s Hospital, School of Medicine, Zhejiang University, Hangzhou, China, CHINA

## Abstract

**Purpose:**

Although current clinical guidelines recommend surgery or radiotherapy for non-bulky IB-IIA cervical cancer, clinical data supporting the curative role of radiotherapy in the early-stage disease are insufficient. We evaluated the prognostic implications of definitive radiotherapy and determined its optimal use in clinical practice.

**Methods:**

Patients with non-bulky (<4 cm) IB-IIA cervical cancer who underwent hysterectomy or primary radiotherapy between 1988 and 2015 were identified from the Surveillance, Epidemiology, and End Results database. Based on the use of brachytherapy and/or chemotherapy, the primary radiotherapy group was classified into three cohorts: hysterectomy vs. radiotherapy overall, with/without brachytherapy and/or chemotherapy (cohort A); radiotherapy and brachytherapy with/without chemotherapy (patients with external beam radiation alone were excluded, cohort B); radiotherapy with brachytherapy and chemotherapy (patients who did not receive chemotherapy were additionally excluded, cohort C). Disease-specific survival (DSS) after hysterectomy was compared to that after primary radiotherapy in each cohort.

**Results:**

Among the 9,391 initially identified patients, 1,762, 1,244, and 750 patients were classified into cohorts A, B, and C, respectively, after propensity score matching. In cohort A, DSS after primary radiotherapy was inferior to that after hysterectomy (*P* = 0.001). In cohort B, a trend toward differential survival in favor of hysterectomy was observed with marginal significance (*P* = 0.061). However, in cohort C, DSS after primary radiotherapy was not significantly different to that after hysterectomy (*P* = 0.127). According to hazard rate function plots, patients receiving external beam radiation alone had an increased short-term risk of disease-specific mortality, whereas patients without evidence of chemotherapy had a distinct late risk surge at approximately 15 years of follow-up.

**Conclusion:**

Optimizing radiotherapy methods with brachytherapy and the use of chemotherapy should be considered for the long-term curative efficacy of primary radiotherapy for non-bulky IB-IIA cervical cancer. Further studies are warranted to corroborate our results.

## Introduction

According to the Global Cancer Observatory 2020 report, cervical cancer is the fourth most commonly diagnosed cancer in women worldwide [1]. Although the incidence rates of cervical cancer have declined, the malignancy remains a challenging problem in lower-middle-income countries [2]. In the updated cancer statistics in the United States, an estimated 13,800 new cases will be diagnosed with carcinoma of the uterine cervix, while 4,290 patients will die of this malignancy [3].

Localized early-stage disease accounts for approximately 44% of cervical cancer cases, and a 5-year survival rate of 92% has been reported in a subset of patients [4]. Surgical resection, including the minimally invasive approach, is the standard management for early-stage (stage IA) tumors, whereas concurrent chemoradiotherapy (CRT) is the initial treatment for bulky (>4 cm) stage IB3 and IIA2 tumors [5]. Furthermore, the National Comprehensive Cancer Network guidelines recommend choosing between radical hysterectomy and primary radiotherapy (RT) for non-bulky IB1, IB2, and IIA1 tumors [6]. The policy suggesting either local treatment option is mainly based on one randomized trial that reported similar survival outcomes with the two local treatment options [7]. In contrast, other observational studies and meta-analyses have consistently reported the superiority of hysterectomy over RT [8–11]. Owing to insufficient high-level evidence, it is uncertain whether these findings are applicable to clinical practice.

Hence, this study aimed to evaluate the prognostic implications of primary RT as definitive therapy and determine its optimal use in non-bulky stage IB-IIA cervical cancer. According to the use of brachytherapy and/or chemotherapy in the primary RT group, the irradiated patients were categorized into three discrete cohorts. After propensity score matching, the long-term survival outcomes of hysterectomy and primary RT were compared. This study provides a better understanding of the therapeutic role of definitive RT for early-stage cervical cancer in the contemporary era.

## Materials and methods

### Patients

After obtaining approval from the Surveillance, Epidemiology, and End Results (SEER) through the Research Data Agreement process [12], this study analyzed the SEER 18 data, the publicly available cancer registry of the National Cancer Institute in the United States [13]. All personal information in the database was classified by assigning numbers to the patients. Since this study retrospectively reviewed the population-based database and used de-identified patient data, informed consent from patients was not required. The data were extracted and manipulated according to the guidelines [14].

The raw data file, including multifarious patient-, tumor-, and treatment-related medical records, was extracted from the case listing session of the SEER*Stat software (version 8.3.6; National Institutes of Health, Bethesda, MD, USA) [15]. Based on the “Site recode ICD-O-3/WHO 2008” variable, the primary tumor site was defined as the cervix uteri. Based on the third revision of the International Classification of Diseases for Oncology (ICD-O-3), the histological type was confirmed using a malignant behavior code. The eligibility criteria were as follows: 1) age ≥18 years; 2) year of diagnosis between 1988 and 2015; 3) no distant metastasis at initial diagnosis; 4) Stage IB1, IB2, or IIA1 tumors according to the revised staging system of the International Federation of Gynecology and Obstetrics (FIGO); 5) primary tumor size ≤4 cm; 6) histology of squamous cell carcinoma, adenocarcinoma, and adenosquamous carcinoma; 7) cancer-directed treatment with surgery (total or radical hysterectomy with/without perioperative treatment) or primary RT (external beam radiotherapy [EBRT], brachytherapy, or a combination of EBRT and brachytherapy, with or without the use of chemotherapy); 8) no previous history of a cancer diagnosis. S1 Fig shows a flowchart of the patient selection process.

### Definition of irradiated patients in the three cohorts

**Fig 1:**
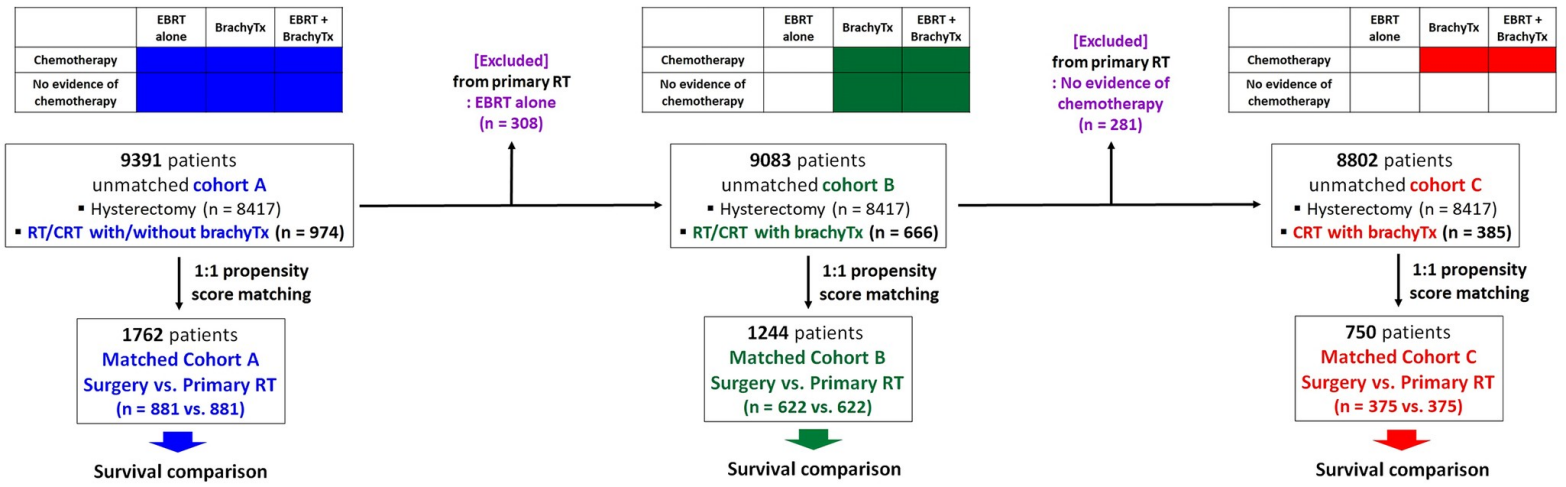
Definition of cohorts A, B, and C and composition of patient subsets before and after propensity score matching. EBRT, external beam radiotherapy; brachyTx, brachytherapy; RT, radiotherapy; CRT, chemoradiotherapy.

To assess the prognostic difference between surgery and primary RT, we categorized patients into three comparison cohorts—cohorts A, B, and C. Fig 1 shows the definition of the three cohorts. Starting from cohort A and going to cohorts B and C, we specified the primary RT group step-by-step. Cohort B was specified, excluding patients with EBRT alone from cohort A, while cohort C was additionally defined, excluding other patients who did not receive chemotherapy from cohort B. Finally, according to the use of brachytherapy and/or chemotherapy, the primary RT groups in cohorts A, B, and C included patients treated with RT or CRT with or without brachytherapy, RT or CRT with brachytherapy, and CRT with brachytherapy, respectively.

### Statistical analysis

Propensity score-adjusted calculations were performed to reduce potential selection bias in the comparison between surgery and primary RT. The propensity score indicates the probability of being assigned to a certain treatment group; hence, baseline covariates were used for the propensity score-adjusted calculation and propensity-score-matched comparison [16]. After calculating the propensity scores using a non-parsimonious logistic regression model, a one-to-one matching process was followed using the nearest-neighbor method, with a caliper of 0.2 without replacement. For each cohort, the final propensity score-matched comparison set with the lowest standardized difference (SD) values was established (acceptable if SD < 0.1) [17].

Baseline characteristics of the surgery and primary RT groups were compared using Pearson’s chi-square test and the Mann-Whitney U test for categorical and continuous variables, respectively. The primary outcome of interest was disease-specific survival (DSS), defined as the time interval between the date of diagnosis and the date of death due to cervical cancer. Before and after propensity score matching in cohorts A, B, and C, long-term DSS outcomes were compared between the surgery and primary RT groups using Kaplan-Meier analysis with the log-rank test. After the assessment of proportional hazards assumptions with log[-log(survival)] plots, the Cox proportional hazards model was used for multivariate analysis of prognostic factors. The threshold for statistical significance was set at *P* <0.05. IBM SPSS Statistics 18 (IBM Corp., Armonk, NY, USA) and R (version 4.0.2; R Foundation for Statistical Computing, Vienna, Austria) were used for all statistical analyses.

## Results

### Study population before and after propensity score matching

Based on the aforementioned eligibility criteria, we initially identified 9,391 patients (Table 1). The median age of the overall population was 44 years (range, 18–97 years). In the study population, 78% of participants were Caucasian, and 51% of them were married. The proportion of squamous cell carcinoma histology (66%) was higher than that of adenocarcinoma (27%) or adenosquamous carcinoma (7%). Regarding primary tumor grade, 41% and 35% of the patients had moderately and poorly differentiated tumors, respectively. Furthermore, 92% and 8% of patients had FIGO stage IB and IIA tumors, respectively, and 51% and 49% had tumors measuring ≤2 cm and >2 cm, respectively. Most patients had a node-negative status (84%) and localized stage (78%). In total, 90% of patients underwent total or radical hysterectomy, and 10% of patients were treated with primary RT, including RT or CRT, with or without brachytherapy.

**Table 1:** Baseline characteristics of the initial study population (N = 9391).

Characteristics	Number of patients (%)
Age (years)	
Median (range)	44 (18–97)
Race	
Caucasian	7366 (78)
African American	897 (10)
Others	1070 (11)
Unknown	58 (1)
Marital status	
Married	4837 (51)
Not married	4214 (45)
Unknown	340 (4)
Histology	
Squamous cell carcinoma	6232 (66)
Adenocarcinoma	2507 (27)
Adenosquamous carcinoma	652 (7)
Tumor grade	
Well differentiated	1104 (12)
Moderately differentiated	3812 (41)
Poorly differentiated	3260 (35)
Undifferentiated	141 (1)
Unknown	1074 (11)
FIGO stage	
IB	8667 (92)
IIA	724 (8)
Tumor size (cm)	
≤ 2cm	4738 (51)
> 2cm	4653 (49)
Lymph node status	
Negative	7922 (84)
Positive	1312 (14)
Unknown	157 (2)
SEER stage	
Localized	7338 (78)
Regional	2053 (22)
Local treatment	
Surgery	8417 (90)
Primary radiotherapy^a^	974 (10)

^a^Radiotherapy or chemoradiotherapy with external beam radiotherapy, brachytherapy, or a combination of both. FIGO, International Federation of Gynecology and Obstetrics; SEER, Surveillance, Epidemiology, and End Results.

Details of the treatment methods are listed in S1 Table. In the hysterectomy group, 65% of patients underwent surgery alone. Surgical evaluation of the lymph nodes was performed in most patients who underwent surgery (90%). In the primary RT group, 32% and 61% of patients underwent EBRT alone and a combination of both, respectively. Chemotherapy was administered to 60% of patients. For primary RT, the proportion of patients treated with brachytherapy gradually declined over time but the proportion of patients treated with chemotherapy increased over time, especially in the more recent years (S2 Fig).

Propensity score matching was performed to compare the surgery and primary RT groups within cohorts A, B, and C. Table 2 lists the propensity score matching of cohort A (hysterectomy vs. RT or CRT overall with or without brachytherapy) based on the available demographic and clinicopathological variables (age, race, marital status, histology, tumor grade, FIGO stage, tumor size, lymph node status, and SEER stage). The matching model was also applied to identify matched cohort B (hysterectomy vs. RT or CRT with brachytherapy; S2 Table) and C (hysterectomy vs. CRT with brachytherapy; S3 Table). Three comparison cohorts of hysterectomy versus primary RT were established: 1,762, 1,244, and 750 patients in cohorts A, B, and C, respectively, after propensity score matching (Fig 1).

**Table 2:** Distribution of baseline variables before and after propensity score matching in cohort A.

Characteristics	Before matching [n (%)]		*Standardized difference*	After matching [n (%)]		*Standardized difference*
	Surgery	Primary RT		Surgery	Primary RT	
	(n = 8417)	(n = 974)		(n = 881)	(n = 881)	
Age (years)						
Mean ± SD	45.3 ± 12.2	56.3 ± 16.2	0.682	54.6 ± 14.4	54.8 ± 15.7	0.016
Race						
White	6645 (79)	721 (74)	0.036	690 (79)	655 (74)	0.037
Black	742 (9)	155 (16)		91 (10)	139 (16)	
Others	975 (11)	95 (10)		97 (11)	84 (10)	
Unknown	55 (1)	3 (0)		3 (0)	3 (0)	
Marital status						
Married	4457 (53)	380 (39)	0.254	381 (43)	362 (41)	0.025
Not married	3655 (43)	559 (57)		461 (52)	487 (55)	
Unknown	305 (4)	35 (4)		39 (5)	32 (4)	
Histology						
Squamous cell carcinoma	5423 (64)	809 (83)	-0.456	731 (83)	724 (82)	0.037
Adenocarcinoma	2378 (28)	129 (13)		124 (14)	122 (14)	
Adenosquamous carcinoma	616 (7)	36 (4)		26 (3)	35 (4)	
Tumor grade						
Well differentiated	1054 (13)	50 (5)	0.463	35 (4)	48 (5)	0.055
Moderately differentiated	3484 (41)	328 (34)		242 (27)	315 (36)	
Poorly differentiated	2952 (35)	308 (32)		451 (51)	290 (33)	
Undifferentiated	128 (1)	13 (1)		24 (3)	12 (1)	
Unknown	799 (10)	275 (28)		129 (15)	216 (25)	
FIGO stage						
IB	7994 (95)	673 (69)	0.560	665 (75)	653 (74)	0.029
IIA	423 (5)	301 (31)		216 (25)	228 (26)	
Tumor size (cm)						
Mean ± SD	2.1 ± 1.1	3.0 ± 1.0	0.829	2.9 ± 1.0	2.9 ± 1.1	0.017
Lymph node status						
Negative	7210 (86)	721 (73)	0.326	634 (72)	655 (74)	0.028
Positive	1141 (13)	171 (18)		218 (25)	160 (18)	
Unknown	66 (1)	91 (9)		29 (3)	66 (8)	
SEER stage						
Localized	6808 (81)	530 (54)	0.531	521 (59)	512 (58)	0.021
Regional	1609 (19)	444 (46)		360 (41)	369 (42)	

RT, radiotherapy; SD, standard deviation; FIGO, International Federation of Gynecology and Obstetrics; SEER, Surveillance, Epidemiology, and End Results.

### Survival comparisons between the hysterectomy and primary RT groups

**Fig 2:**
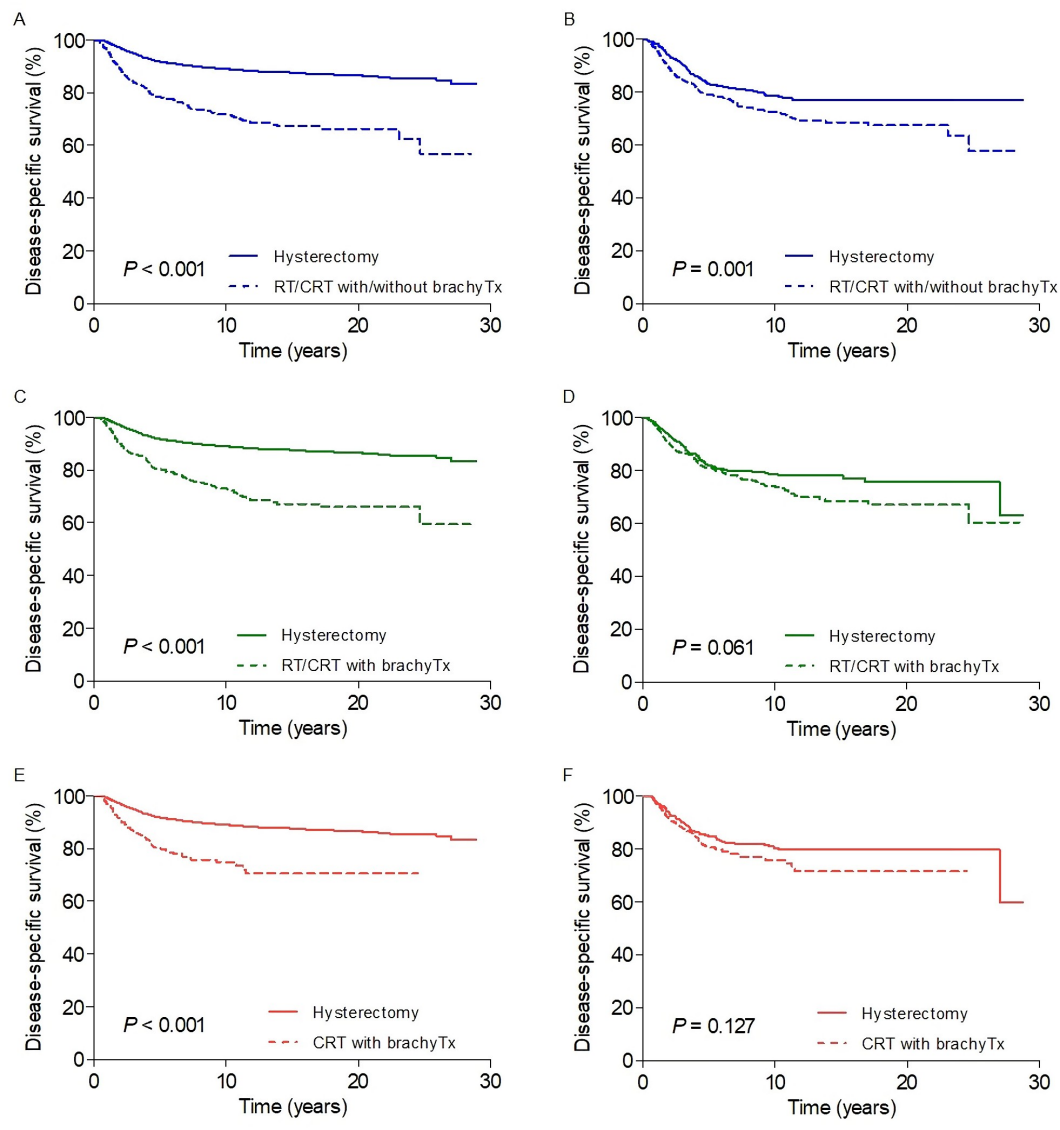
Disease-specific survival according to hysterectomy and primary radiotherapy (A, C, E) before and (B, D, F) after propensity score matching in cohorts A (*blue*), B (*green*), and C (*red*), respectively. RT, radiotherapy; CRT, chemoradiotherapy; brachyTx, brachytherapy.

Fig 2 presents the DSS curves according to the surgery and primary RT. Before propensity score matching, the 20-year DSS rates in the hysterectomy group were better than those in the primary RT group for all cohorts (87% vs. 66%, 66%, and 71% in cohorts A, B, and C, respectively; *P* < 0.001 for all comparisons), and significant differences were also observed in terms of overall survival (*P* < 0.001 for all comparisons; S3 Fig). The comparison of DSS in matched cohort A also indicated statistical significance in favor of surgery (*P* = 0.001), whereas a trend toward differential survival outcomes was observed with marginal significance in matched cohort B (*P* = 0.061). In matched cohort C, however, no significant difference in DSS was observed between the hysterectomy and primary RT groups (*P* = 0.127). The results of univariate analyses of DSS in cohorts A, B, and C are presented in S4 Table.

Table 3 shows the results of the Cox regression analysis in the matched cohorts A, B, and C. In cohort A, FIGO stage IIA (vs. IB; *P* = 0.003), tumor size >2 cm (vs. ≤2 cm; *P* < 0.001), node-positive status (vs. node-negative status; *P* < 0.001), and primary RT (vs. surgery; *P* = 0.001) were significantly associated with poor DSS. In cohort B, FIGO stage IIA (*P* = 0.027), tumor size >2 cm (*P* = 0.006), and node-positive status (*P* = 0.013) were significantly associated with poor prognosis; however, only marginal significance was observed when comparing the surgery and primary RT groups (*P* = 0.055). Multivariate analysis of cohort C showed that tumor grade III-IV (*P* = 0.006) and node-positive status (*P* = 0.033) were factors independently associated with DSS.

**Table 3:** Prognostic factors associated with disease-specific survival.

Variables	Cohort A		*P*	Cohort B		*P*	Cohort C		*P*
	HR	95% CI		HR	95% CI		HR	95% CI	
Age (years)^a^									
<54	Ref								
≥54	1.23	0.99–1.53	0.068						
Age (years)^b^									
<50							Ref		
≥50							1.34	0.90–2.00	0.156
Race									
Caucasian	Ref								
Others	1.25	0.99–1.59	0.066						
Tumor grade									
I–II				Ref			Ref		
III–IV				1.35	0.99–1.84	0.055	1.84	1.20–2.84	0.006
FIGO stage									
IB	Ref			Ref			Ref		
IIA	1.42	1.13–1.80	0.003	1.40	1.04–1.90	0.027	1.36	0.90–2.05	0.145
Tumor size (cm)									
≤2 cm	Ref			Ref			Ref		
>2 cm	1.87	1.36–2.57	< 0.001	1.92	1.21–3.04	0.006	1.34	0.70–2.54	0.378
Lymph node status									
Negative	Ref			Ref			Ref		
Positive	1.74	1.37–2.22	< 0.001	1.50	1.09–2.08	0.013	1.56	1.04–2.35	0.033
Local treatment									
Surgery	Ref			Ref					
Primary RT^c^	1.45	1.16–1.81	0.001	1.34	0.99–1.81	0.055			

^a^The median value in cohorts A and B was used as the optimal cutoff.

^b^The median value in the cohort C was used as an optimal cutoff.

^c^RT or CRT overall with/without brachytherapy in cohort A; RT or CRT with brachytherapy in cohort B; CRT with brachytherapy in cohort C. HR, hazard ratio; CI, confidence interval; Ref, reference; FIGO, International Federation of Gynecology and Obstetrics; RT, radiotherapy; CRT, chemoradiotherapy.

### Time-course risks of disease-specific mortality after primary RT

**Fig 3:**
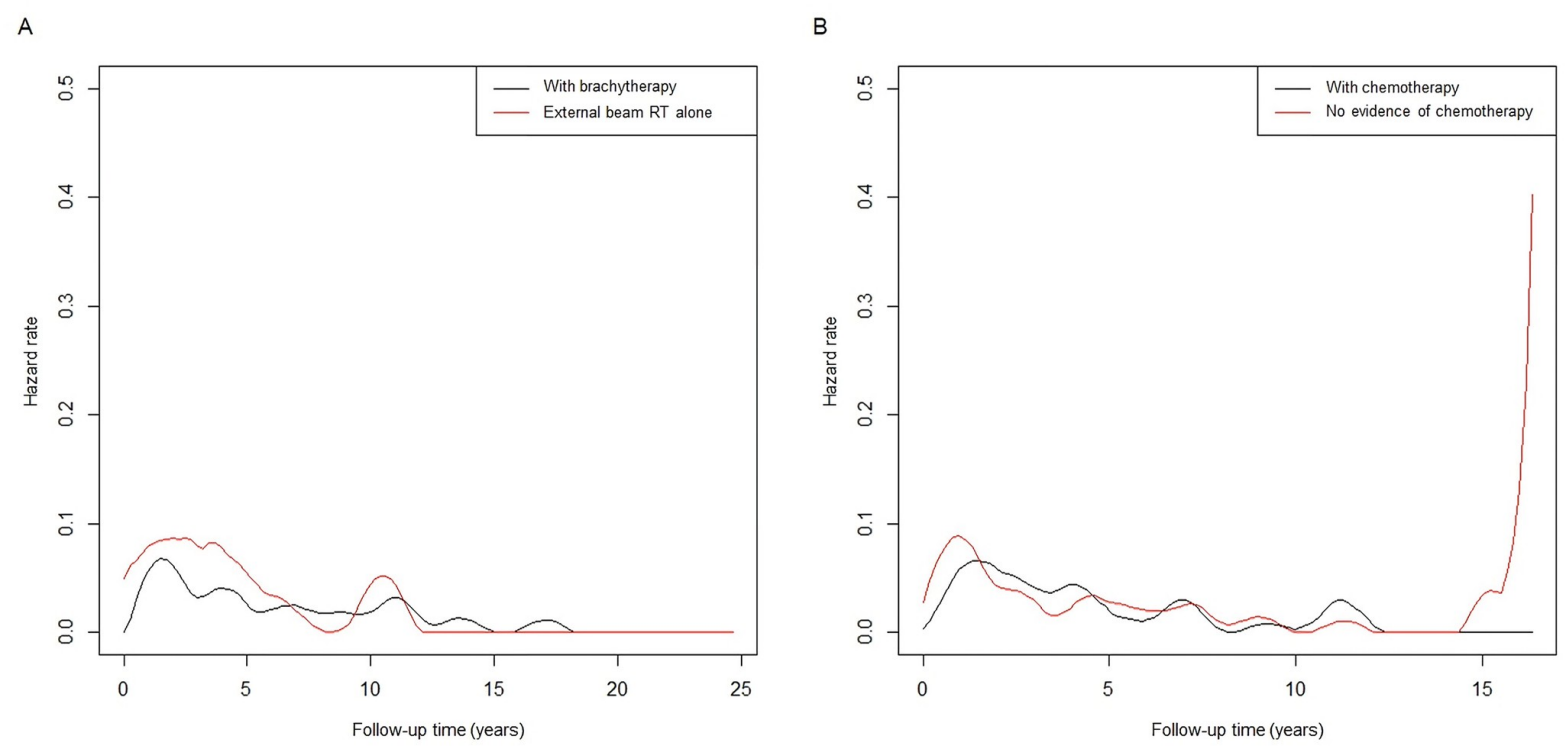
Hazard rate function plots of disease-specific mortality in patients who underwent primary radiotherapy according to use of (A) brachytherapy and (B) chemotherapy. RT, radiotherapy.

Fig 3 shows the time-course hazard rate function plots of disease-specific mortality of the primary RT group in the initial study population (n = 974). EBRT alone increased the short-term risk of disease-specific mortality over the 5-year follow-up period. A distinct late risk surge was observed at approximately 15 years of follow-up in patients without evidence of chemotherapy, but not in patients who received chemotherapy.

## Discussion

To evaluate the prognostic implications of definitive RT in non-bulky stage IB-IIA cervical cancer, propensity score-adjusted analyses of the three cohorts were performed. In cohort A, hysterectomy was better than RT or CRT overall with or without brachytherapy. In cohort B, excluding patients treated with EBRT alone, the comparison between hysterectomy and RT or CRT with brachytherapy showed a trend toward improved survival after surgery, with marginal significance. The primary RT group in cohort C was confined to patients who received CRT with brachytherapy, and there was no significant prognostic difference compared to the hysterectomy group. Primary RT with EBRT alone led to increased short-term risks of disease-specific mortality over a 5-year follow-up period, whereas a distinct late risk surge was observed at approximately 15 years of follow-up in patients without evidence of chemotherapy. Based on the step-by-step matching and survival comparisons, this study suggests the need for brachytherapy and chemotherapy for the long-term curative effect of primary RT in non-bulky early-stage cervical cancer.

Only one randomized controlled trial compared surgery and primary RT in this category of patients [7]. The 5-year rates of overall survival and disease-free survival in the primary RT group were comparable to those in the surgery group (83% for both and 74% for both), and the results have been applied to current clinical guidelines. More recently, a study demonstrated that similar survival outcomes between the two treatment modalities were also valid in the 20-year data (*P* = 0.280) [18]. However, approximately one-third of the patients enrolled in the trial had bulky tumors (>4 cm), designated as stage IB3 or IIA2, based on the current staging system. Definitive CRT, rather than primary surgery, is the standard treatment for bulky tumors; hence, the potential survival benefit of primary RT might be overestimated in the data. Furthermore, other retrospective studies suggesting comparable outcomes between surgery and primary RT also included a substantial proportion of stage IIB or bulky stage IB tumors [19,20].

Given the absence of other prospective data, several population-based studies have been reported [8,9,21]. Brewster et al. initially analyzed patients with stage IB-IIA cervical cancer from the SEER database (1988–1995) [9]. The 5-year survival outcomes favored hysterectomy in tumors measuring ≤4 cm; however, there was no significant prognostic difference between hysterectomy and RT in tumors measuring >4 cm. Later, Bansal et al. updated the data (1988–2005) [8], suggesting that hysterectomy led to improved overall survival in patients with tumors measuring <4 cm and 4–6 cm but not in patients with tumors measuring >6 cm. These outcomes in favor of hysterectomy for tumors measuring <4 cm were consistent with the results of cohort A in our study. However, we conducted propensity-score-matched comparisons of the three discrete cohorts. After excluding patients treated with EBRT alone (cohort B) and patients without evidence of chemotherapy (cohort C) from cohort A, the subsequent survival comparisons suggested that the appropriate use of brachytherapy and chemotherapy is required to achieve the curative aim of primary RT.

More recently, some studies evaluated the prognostic difference between hysterectomy and primary CRT [10,21,22]. The most recent study was a Chinese study that analyzed pooled data on patients with stage IB1-IIA2 cervical cancer (2004–2016) from 37 hospitals [22]. The matched comparison revealed a better prognosis after hysterectomy than after RT, which was inconsistent with our results. However, the Chinese study had several limitations, such as the inclusion of a considerable proportion of bulky tumors, use of a smaller number of covariates in the matching process, and the absence of multivariate analysis data. Our results of the matched comparisons demonstrated that the superiority of hysterectomy to RT was gradually attenuated in cohorts B and C. In addition to the time-course hazard rates, comparative survival analyses based on the discrete matched cohorts underlined the therapeutic relevance of brachytherapy and chemotherapy along with primary RT in non-bulky IB-IIA cervical cancer.

The increased use of EBRT alone in recent years might be attributable to technical advances in the contemporary RT era [23]. Given the widespread use of high-precision conformal techniques, dosimetric profiles using intensity-modulated RT, proton therapy, and stereotactic body RT have been investigated to clarify whether advanced RT modalities could be alternatives to brachytherapy [24–26]. Nevertheless, the conformality to direct a higher radiation dose to the primary tumor and effective sparing of neighboring organs were prominent in brachytherapy planning, suggesting that brachytherapy did not appear to be easily replaced by EBRT alone [26]. A prior SEER analysis also demonstrated that brachytherapy is an integral part of complete tumor eradication and improves survival in cervical cancer [27]. Furthermore, our hazard rate function plots showed a distinguishable risk surge in patients without evidence of chemotherapy, even after the 15-year follow-up. Despite the role of chemotherapy in controlling the latent systemic tumor burden, its prognostic association has not been well studied in non-bulky early-stage cervical cancer. We demonstrated that the application of brachytherapy and chemotherapy is a substantial element in primary RT to obtain therapeutic efficacy comparable to that of hysterectomy.

Based on the pros and cons of surgery and primary RT, age, medical comorbidities, fertility status, accuracy of clinical staging, and treatment-related complications are major factors for consideration in practice. For young patients with smaller tumors, the fertility-sparing surgical approach is preferred over primary RT [5]. Surgical resection is also preferentially recommended in lower-middle-income countries where advanced imaging tools or RT modalities are not available [28]. However, primary RT is the first treatment option for patients with medical comorbidities that increase the risk associated with general anesthesia. In this study, we demonstrate that primary RT, as an initial definitive therapy, needs to be considered in clinical settings, where the administration of brachytherapy and chemotherapy is sufficiently feasible.

This study has several limitations. Despite the propensity score-adjusted analyses, potential selection bias was not completely eliminated in the retrospective design. The general medical condition could not be considered in the matching process due to the lack of information; thus, we mainly analyzed DSS data as the primary outcome of interest rather than overall survival. Further, detailed information on RT methods (total radiation dose, daily fraction size, extent of the radiation field, and techniques) or chemotherapy (regimen and cycles) was not available in the database. Technical aspects can affect the clinical outcomes of primary RT. Since the quality of RT methods was not confirmed, treatment benefits in the primary RT group might be partially underestimated in comparison with radical surgery. The appropriate or inappropriate administration of perioperative treatment after surgery was not assessed because the surgical margin status, such as R0, R1, and R2, was unknown. The sequence of the two treatments was not indicated in the database for the combined use of chemotherapy and primary RT. Recent advances in RT techniques were not partially reflected because some patients were treated a few decades ago. Nevertheless, the SEER program is a representative nationwide registry that allows access to large-scale real-world data in practice.

In this study, we evaluated the effectiveness and optimal use of primary RT as definitive therapy for non-bulky early-stage cervical cancer. Although hysterectomy led to better survival outcomes than RT in the overall study population, the survival difference was reduced and attenuated when the primary RT group was confined to patients treated with brachytherapy and chemotherapy. Considering the time-course risk patterns in hazard rate function plots, the appropriate use of brachytherapy and chemotherapy with primary RT would contribute to reducing the short- and long-term risks of disease-specific mortality, respectively. Thus, we suggest that primary RT methods need to be optimized with regard to brachytherapy and chemotherapy to obtain long-term curative efficacy in patients with non-bulky early-stage cervical cancer. Further prospective studies are required to corroborate our results.

## Supporting information

S1 Fig Flowchart of the patient selection process. FIGO, International Federation of Gynecology and Obstetrics.
(TIF)

S2 Fig Changes in the rates of (A) irradiation methods and (B) use of chemotherapy over time in the primary radiotherapy group. (TIF)

S3 Fig Overall survival according to hysterectomy and primary radiotherapy of (A) cohort A, (B) cohort B, and (C) cohort C before propensity score matching. RT, radiotherapy; CRT, chemoradiotherapy; brachyTx, brachytherapy.
(TIF)

S1 Table Details of treatment methods. (DOCX)

S2 Table Distribution of baseline variables before and after propensity score matching in cohort B. (DOCX)

S3 Table Distribution of baseline variables before and after propensity score matching in cohort C. (DOCX)

S4 Table Univariate analysis of the matched cohorts A, B, and C. (DOCX)

S1 Rawdata (ZIP)
