# Supplementary figures and images for: Optimal use of radiotherapy in the definitive treatment of non-bulky IB–IIA cervical cancer: A population-based long-term survival analysis
Source: PLoS One. 2021 Jun 24;16(6):e0253649. doi: 10.1371/journal.pone.0253649 (PMC8224971; doi:10.1371/journal.pone.0253649)

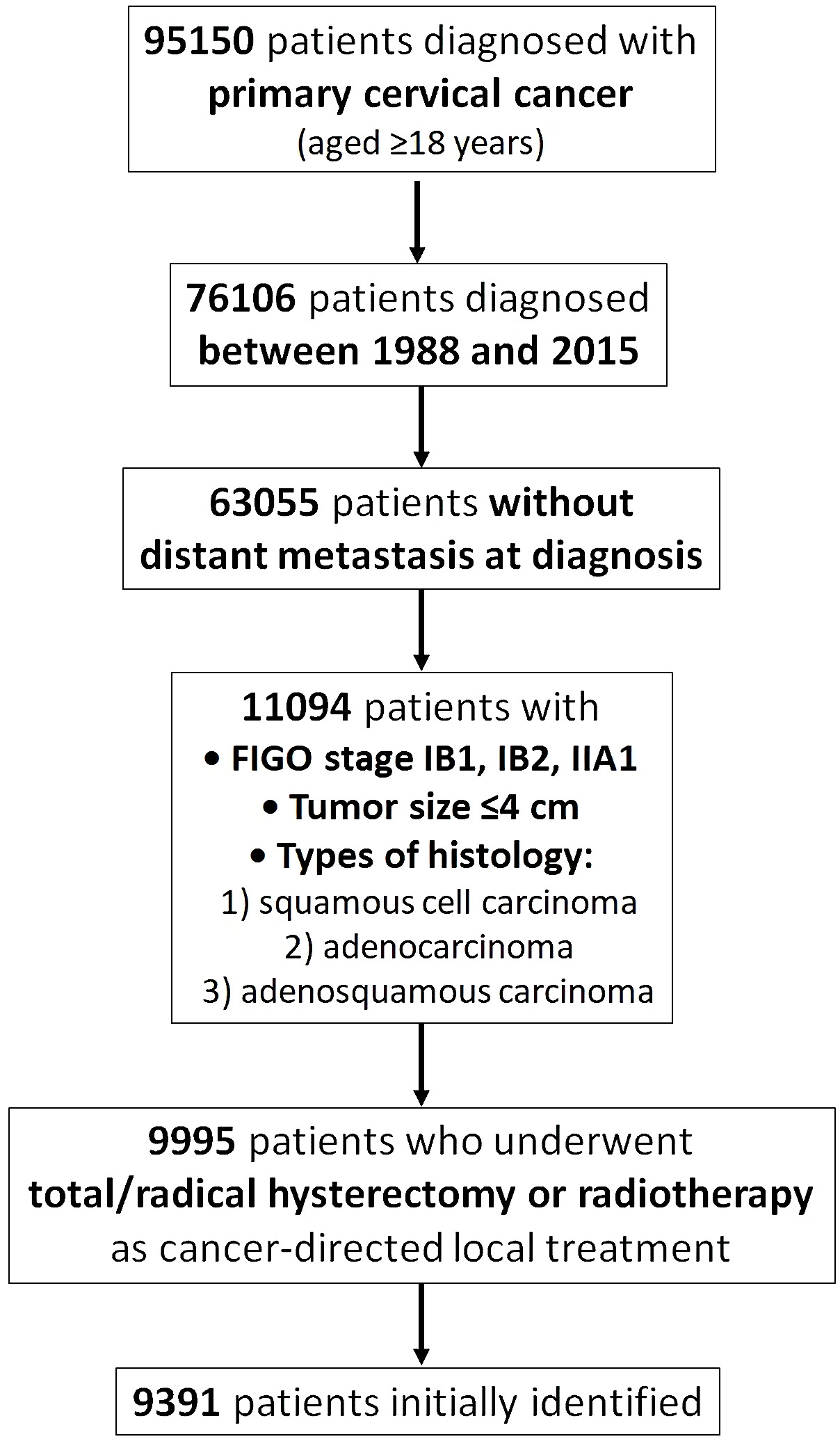

Supplement: S1 Fig — FIGO, International Federation of Gynecology and Obstetrics. (TIF) [file pone.0253649.s001.tif]

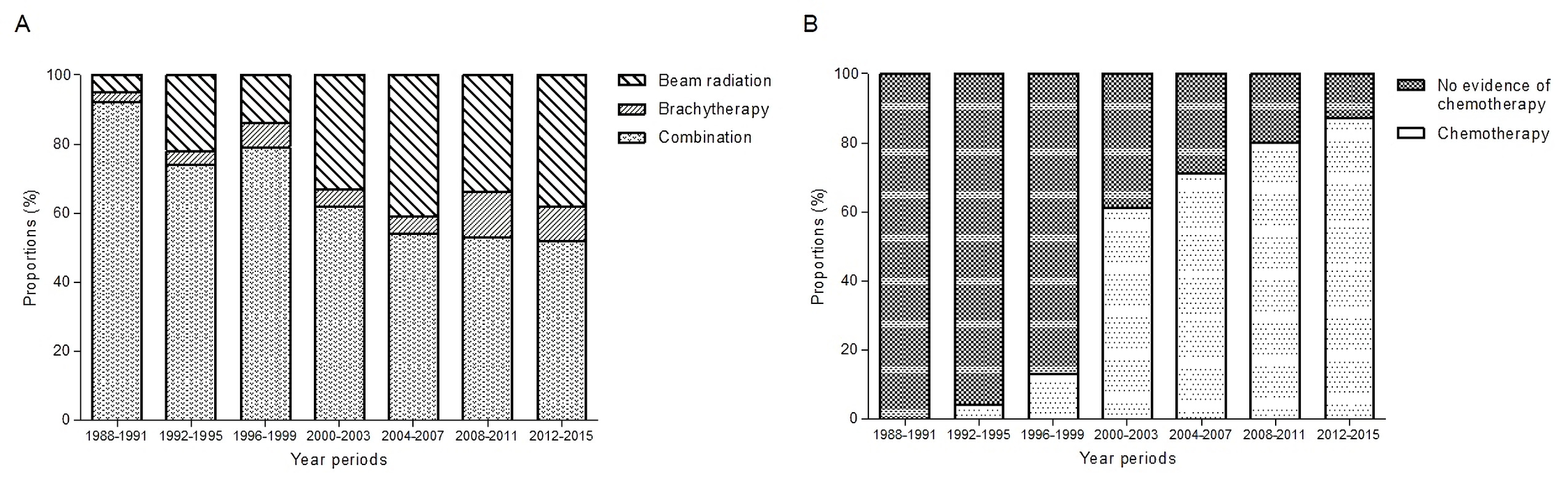

Supplement: S2 Fig — (TIF) [file pone.0253649.s002.tif]

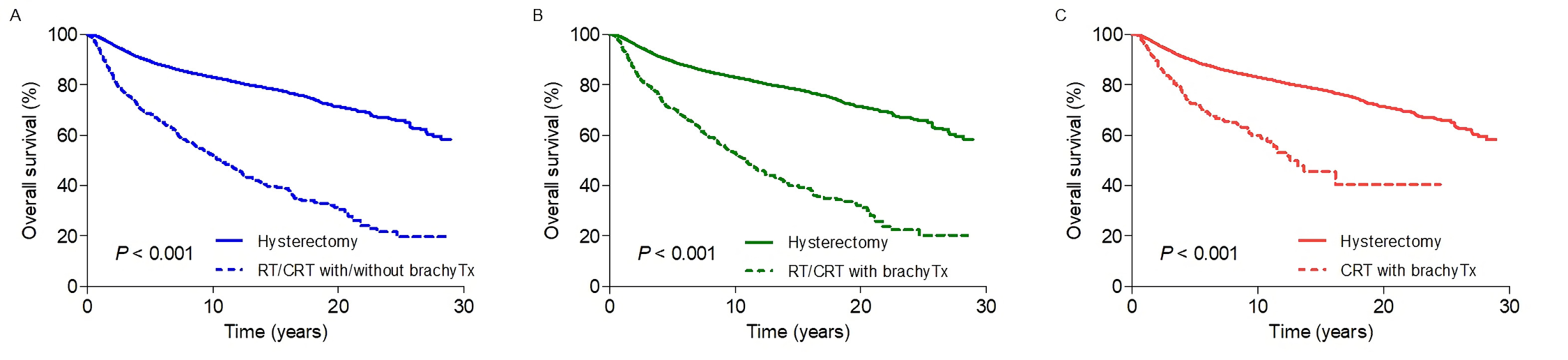

Supplement: S3 Fig — RT, radiotherapy; CRT, chemoradiotherapy; brachyTx, brachytherapy. (TIF) [file pone.0253649.s003.tif]
