# Supplementary material for: Optimal use of radiotherapy in the definitive treatment of non-bulky IB–IIA cervical cancer: A population-based long-term survival analysis
Source: PLoS One. 2021 Jun 24;16(6):e0253649. doi: 10.1371/journal.pone.0253649 (PMC8224971; doi:10.1371/journal.pone.0253649)
Supplement: S1 Table — (DOCX) [file pone.0253649.s004.docx]

**S1 Table.** Details of treatment methods.

| Treatment characteristics | Number of patients (%) | |
| --- | --- | --- |
|  | Surgery | Primary RT |
|  | (*n* = 8417) | (*n* = 974) |
| Perioperative treatment |  |  |
| Surgery alone | 5480 (65) |  |
| Surgery with chemotherapy | 117 (1) |  |
| Surgery with RT | 1338 (16) |  |
| Surgery with chemoradiotherapy | 1482 (18) |  |
| Surgical evaluation of lymph nodes |  |  |
| Yes | 7552 (90) |  |
| No | 856 (10) |  |
| Unknown | 9 (0) |  |
| Types of irradiation |  |  |
| EBRT |  | 308 (32) |
| Brachytherapy |  | 72 (7) |
| EBRT and brachytherapy |  | 594 (61) |
| Chemotherapy combined with RT |  |  |
| No |  | 394 (41) |
| Yes |  | 580 (60) |

RT, radiotherapy; EBRT, external beam radiotherapy.
