# Supplementary material for: Optimal use of radiotherapy in the definitive treatment of non-bulky IB–IIA cervical cancer: A population-based long-term survival analysis
Source: PLoS One. 2021 Jun 24;16(6):e0253649. doi: 10.1371/journal.pone.0253649 (PMC8224971; doi:10.1371/journal.pone.0253649)
Supplement: S2 Table — (DOCX) [file pone.0253649.s005.docx]

**S2 Table.** Distribution of baseline variables before and after propensity score matching in cohort B.

| Characteristics | Before matching [n (%)] | | *Standardized*  *difference* | After matching [n (%)] | | *Standardized*  *difference* |
| --- | --- | --- | --- | --- | --- | --- |
|  | Surgery | Primary RT |  | Surgery | Primary RT |  |
|  | (n = 8417) | (n = 666) |  | (n = 622) | (n = 622) |  |
| Age (years) |  |  |  |  |  |  |
| Mean ± SD | 45.3 ± 12.2 | 55.7 ± 15.7 | 0.665 | 54.4 ± 14.5 | 54.7 ± 15.5 | 0.017 |
| Race |  |  |  |  |  |  |
| White | 6645 (79) | 488 (73) | 0.054 | 482 (78) | 457 (73) | 0.005 |
| Black | 742 (9) | 106 (16) |  | 57 (9) | 100 (16) |  |
| Others | 975 (11) | 72 (11) |  | 78 (12) | 54 (11) |  |
| Unknown | 55 (1) | 0 (0) |  | 5 (1) | 0 (0) |  |
| Marital status |  |  |  |  |  |  |
| Married | 4457 (53) | 268 (40) | 0.225 | 260 (42) | 257 (41) | -0.015 |
| Not married | 3655 (43) | 377 (57) |  | 334 (54) | 345 (56) |  |
| Unknown | 305 (4) | 21 (3) |  | 28 (4) | 20 (3) |  |
| Histology |  |  |  |  |  |  |
| Squamous cell carcinoma | 5423 (65) | 553 (83) | -0.460 | 513 (83) | 512 (82) | 0.010 |
| Adenocarcinoma | 2378 (28) | 89 (13) |  | 88 (14) | 87 (14) |  |
| Adenosquamous carcinoma | 616 (7) | 24 (4) |  | 21 (3) | 23 (4) |  |
| Tumor grade |  |  |  |  |  |  |
| Well differentiated | 1054 (13) | 34 (5) | 0.460 | 19 (3) | 33 (5) | 0.033 |
| Moderately differentiated | 3484 (41) | 221 (33) |  | 172 (28) | 215 (35) |  |
| Poorly differentiated | 2952 (35) | 220 (33) |  | 309 (49) | 209 (34) |  |
| Undifferentiated | 128 (2) | 7 (1) |  | 18 (3) | 7 (1) |  |
| Unknown | 799 (9) | 184 (28) |  | 104 (17) | 158 (25) |  |
| FIGO stage |  |  |  |  |  |  |
| IB | 7994 (95) | 452 (68) | 0.580 | 442 (71) | 444 (71) | -0.007 |
| IIA | 423 (5) | 214 (32) |  | 180 (29) | 178 (29) |  |
| Tumor size (cm) |  |  |  |  |  |  |
| Mean ± SD | 2.1 ± 1.1 | 3.0 ± 1.0 | 0.883 | 3.0 ± 0.9 | 3.0 ± 1.0 | 0.025 |
| Lymph node status |  |  |  |  |  |  |
| Negative | 7210 (86) | 486 (73) | 0.338 | 436 (70) | 461 (74) | 0.007 |
| Positive | 1141 (13) | 108 (16) |  | 158 (25) | 105 (17) |  |
| Unknown | 66 (1) | 72 (11) |  | 28 (5) | 56 (9) |  |
| SEER stage |  |  |  |  |  |  |
| Localized | 6808 (81) | 368 (55) | 0.515 | 350 (56) | 360 (58) | -0.032 |
| Regional | 1609 (19) | 298 (45) |  | 272 (44) | 262 (42) |  |

RT, radiotherapy; SD, standard deviation; FIGO, International Federation of Gynecology and Obstetrics; SEER, Surveillance, Epidemiology, and End Results.
