# Supplementary material for: Optimal use of radiotherapy in the definitive treatment of non-bulky IB–IIA cervical cancer: A population-based long-term survival analysis
Source: PLoS One. 2021 Jun 24;16(6):e0253649. doi: 10.1371/journal.pone.0253649 (PMC8224971; doi:10.1371/journal.pone.0253649)
Supplement: S3 Table — (DOCX) [file pone.0253649.s006.docx]

**S3 Table.** Distribution of baseline variables before and after propensity score matching in cohort C.

| Characteristics | Before matching [n (%)] | | *Standardized*  *difference* | After matching [n (%)] | | *Standardized*  *difference* |
| --- | --- | --- | --- | --- | --- | --- |
|  | Surgery  (n = 8417) | Primary RT  (n = 385) |  | Surgery  (n = 375) | Primary RT  (n = 375) |  |
| Age (years) |  |  |  |  |  |  |
| Mean ± SD | 45.3 ± 12.2 | 52.1 ± 14.7 | 0.467 | 51.0 ± 14.4 | 51.6 ± 14.5 | 0.060 |
| Race |  |  |  |  |  |  |
| White | 6645 (79) | 295 (77) | -0.050 | 297 (79) | 286 (76) | 0.013 |
| Black | 742 (9) | 61 (16) |  | 42 (11) | 60 (16) |  |
| Others | 975 (11) | 29 (7) |  | 35 (10) | 29 (8) |  |
| Unknown | 55 (1) |  |  | 1 (0) | 0 (0) |  |
| Marital status |  |  |  |  |  |  |
| Married | 4457 (53) | 161 (42) | 0.197 | 162 (43) | 160 (43) | -0.034 |
| Not married | 3655 (43) | 211 (55) |  | 191 (51) | 202 (54) |  |
| Unknown | 305 (4) | 13 (3) |  | 22 (6) | 13 (3) |  |
| Histology |  |  |  |  |  |  |
| Squamous cell carcinoma | 5423 (65) | 318 (83) | -0.424 | 306 (82) | 308 (82) | -0.011 |
| Adenocarcinoma | 2378 (28) | 51 (13) |  | 53 (14) | 51 (14) |  |
| Adenosquamous carcinoma | 616 (7) | 16 (4) |  | 16 (4) | 16 (4) |  |
| Tumor grade |  |  |  |  |  |  |
| Well differentiated | 1054 (12) | 15 (4) | 0.451 | 11 (3) | 15 (4) | 0.041 |
| Moderately differentiated | 3484 (41) | 134 (35) |  | 105 (28) | 134 (36) |  |
| Poorly differentiated | 2952 (35) | 133 (34) |  | 190 (51) | 133 (35) |  |
| Undifferentiated | 128 (2) | 3 (1) |  | 11 (3) | 3 (1) |  |
| Unknown | 799 (10) | 100 (26) |  | 58 (15) | 90 (24) |  |
| FIGO stage |  |  |  |  |  |  |
| IB | 7994 (95) | 265 (69) | 0.564 | 272 (72) | 265 (71) | 0.040 |
| IIA | 423 (5) | 120 (31) |  | 103 (28) | 110 (29) |  |
| Tumor size (cm) |  |  |  |  |  |  |
| Mean ± SD | 2.1 ± 1.1 | 3.1 ± 1.0 | 1.037 | 3.1 ± 0.9 | 3.1 ± 1.0 | 0.013 |
| Lymph node status |  |  |  |  |  |  |
| Negative | 7210 (86) | 275 (71) | 0.313 | 258 (69) | 268 (71) | -0.030 |
| Positive | 1141 (13) | 98 (26) |  | 110 (29) | 96 (26) |  |
| Unknown | 66 (1) | 12 (3) |  | 7 (2) | 11 (3) |  |
| SEER stage |  |  |  |  |  |  |
| Localized | 6808 (81) | 186 (48) | 0.651 | 179 (48) | 186 (50) | -0.037 |
| Regional | 1609 (19) | 199 (52) |  | 196 (52) | 189 (50) |  |

RT, radiotherapy; SD, standard deviation; FIGO, International Federation of Gynecology and Obstetrics; SEER, Surveillance, Epidemiology, and End Results.
