# Supplementary material for: Optimal use of radiotherapy in the definitive treatment of non-bulky IB–IIA cervical cancer: A population-based long-term survival analysis
Source: PLoS One. 2021 Jun 24;16(6):e0253649. doi: 10.1371/journal.pone.0253649 (PMC8224971; doi:10.1371/journal.pone.0253649)
Supplement: S4 Table — (DOCX) [file pone.0253649.s007.docx]

**S4 Table.** Univariate analysis of the matched cohorts A, B, and C.

| Variables | Cohort A | | *P* | Cohort B | | *P* | Cohort C | | *P* |
| --- | --- | --- | --- | --- | --- | --- | --- | --- | --- |
|  | n (%) | 15-year rate (%) |  | n (%) | 15-year rate (%) |  | n (%) | 15-year rate (%) |  |
| Age (years)^a^ |  |  |  |  |  |  |  |  |  |
| < 54 | 859 (49) | 76 | 0.068 | 604 (49) | 77 | 0.141 |  |  |  |
| ≥ 54 | 903 (51) | 70 |  | 640 (51) | 69 |  |  |  |  |
| Age (years)^b^ |  |  |  |  |  |  |  |  |  |
| < 50 |  |  |  |  |  |  | 362 (48) | 82 | 0.073 |
| ≥ 50 |  |  |  |  |  |  | 388 (52) | 70 |  |
| Race |  |  |  |  |  |  |  |  |  |
| Caucasian | 1345 (76) | 75 | 0.011 | 939 (76) | 73 | 0.337 | 583 (78) | 78 | 0.239 |
| Others | 411 (23) | 68 |  | 300 (24) | 71 |  | 166 (22) | 70 |  |
| Marital status |  |  |  |  |  |  |  |  |  |
| Married | 743 (42) | 74 | 0.466 | 517 (42) | 74 | 0.797 | 322 (43) | 77 | 0.279 |
| Not married | 948 (54) | 71 |  | 679 (55) | 72 |  | 393 (52) | 73 |  |
| Histology |  |  |  |  |  |  |  |  |  |
| Squamous cell carcinoma | 1455 (83) | 74 | 0.269 | 1025 (82) | 74 | 0.234 | 614 (82) | 76 | 0.684 |
| Adenocarcinoma | 246 (14) | 68 |  | 175 (14) | 68 |  | 104 (14) | 78 |  |
| Adenosquamous carcinoma | 61 (3) | 77 |  | 44 (4) | 72 |  | 32 (4) | 85 |  |
| Tumor grade |  |  |  |  |  |  |  |  |  |
| I−II | 640 (36) | 74 | 0.153 | 439 (35) | 75 | 0.069 | 265 (35) | 82 | 0.002 |
| III−IV | 777 (44) | 71 |  | 543 (44) | 72 |  | 337 (45) | 73 |  |
| FIGO stage |  |  |  |  |  |  |  |  |  |
| IB | 1318 (75) | 76 | < 0.001 | 886 (71) | 77 | < 0.001 | 537 (72) | 80 | 0.018 |
| IIA | 444 (25) | 63 |  | 358 (29) | 64 |  | 213 (28) | 67 |  |
| Tumor size (cm) |  |  |  |  |  |  |  |  |  |
| ≤ 2cm | 409 (23) | 81 | < 0.001 | 276 (22) | 82 | < 0.001 | 127 (17) | 86 | 0.008 |
| > 2cm | 1353 (77) | 70 |  | 968 (78) | 71 |  | 623 (83) | 74 |  |
| Lymph node status |  |  |  |  |  |  |  |  |  |
| Negative | 1289 (73) | 76 | < 0.001 | 897 (72) | 76 | < 0.001 | 526 (70) | 80 | 0.002 |
| Positive | 378 (22) | 64 |  | 263 (21) | 65 |  | 206 (28) | 67 |  |
| Chemotherapy |  |  |  |  |  |  |  |  |  |
| None/unknown | 966 (55) | 74 | 0.601 | 693 (56) | 74 | 0.544 | 252 (34) | 81 | 0.088 |
| Yes | 796 (45) | 73 |  | 551 (44) | 73 |  | 498 (66) | 73 |  |
| Local treatment |  |  |  |  |  |  |  |  |  |
| Surgery | 881 (50) | 77 | 0.001 | 622 (50) | 78 | 0.061 | 375 (50) | 80 | 0.127 |
| Primary RT^c^ | 881 (50) | 69 |  | 622 (50) | 68 |  | 375 (50) | 72 |  |

^a^The median value of cohorts A and B was used as an optimal cutoff.

^b^The median value of cohort C was used as an optimal cutoff.

^c^RT or CRT overall with/without brachytherapy in cohort A; RT or CRT with brachytherapy in cohort B; CRT with brachytherapy in cohort C.

FIGO, International Federation of Gynecology and Obstetrics; RT, radiotherapy; CRT, chemoradiotherapy.
